# Conflict-free joint decision by lag and zero-lag synchronization in laser network

**DOI:** 10.1038/s41598-024-54491-1

**Published:** 2024-02-22

**Authors:** Hisako Ito, Takatomo Mihana, Ryoichi Horisaki, Makoto Naruse

**Affiliations:** Department of Information Physics and Computing, Graduate School of Information Science and Technology, The University of Tokyo, 7-3-1 Hongo, Bunkyo-ku, Tokyo, 113-8656 Japan

**Keywords:** Fibre optics and optical communications, Computer science

## Abstract

With the end of Moore’s Law and the increasing demand for computing, photonic accelerators are garnering considerable attention. This is due to the physical characteristics of light, such as high bandwidth and multiplicity, and the various synchronization phenomena that emerge in the realm of laser physics. These factors come into play as computer performance approaches its limits. In this study, we explore the application of a laser network, acting as a photonic accelerator, to the competitive multi-armed bandit problem. In this context, conflict avoidance is key to maximizing environmental rewards. We experimentally demonstrate cooperative decision-making using zero-lag and lag synchronization within a network of four semiconductor lasers. Lag synchronization of chaos realizes effective decision-making and zero-lag synchronization is responsible for the realization of the collision avoidance function. We experimentally verified a low collision rate and high reward in a fundamental 2-player, 2-slot scenario, and showed the scalability of this system. This system architecture opens up new possibilities for intelligent functionalities in laser dynamics.

## Introduction

At the dawn of the 21st century, Moore’s Law^1^, which predicted consistent improvements in microprocessor performance, was nearing its limit, prompting the exploration of new computing frameworks^2,3^. In particular, the demand for computational power has been increasing dramatically with the use of graphics processing units (GPUs). As such, GPUs can be viewed as accelerators in computing. Photonic accelerators^4^ attempt to solve specific problems and take advantage of the high speed and multiplicity offered by using light. In recent years, there have been many studies that apply optics to computational tasks found in photonic accelerators, such as matrix multiplication^5,6^, reservoir computing^7,8^, neural networks^9–12^, neuromorphic computing^13^, reinforcement learning^14,15^, coherent Ising machines^16^, and decision-making^17–22^.

Decision-making in the realm of the multi-armed bandit (MAB) problem is a complex task. This problem, first described by Robbins in 1952^23^, involves choosing from multiple slot machines, each with an unknown probability of winning, with the aim of maximizing profit^24^. In this scenario, excessive exploration could result in missed opportunities for winning, while an over-reliance on prior results might lead to the neglect of a potentially profitable machine. As such, successful decision-making requires a delicate balance between exploration and exploitation^25,26^. Several decision-making strategies for the MAB problem have been demonstrated using photonic principles, leveraging technologies such as single photons^17^, chaotic laser^18,27^, and ring lasers^28^. The competitive multi-armed bandit (CMAB) problem takes the MAB problem a step further by considering scenarios involving multiple players^29^. This problem encompasses a wide range of issues, including resource competition scenarios like network connection problems^30^. As automation and mechanization continue to evolve and become more widespread, the significance of the CMAB problem grows accordingly. In the MAB problem, effective exploration is needed, in addition to this, there is another consideration needs to be taken into account for reward maximization in the CMAB problem. In CMAB scenarios, the rewards are divided due to conflicting choices, necessitating conflict-free, cooperative decision-making to optimize both individual and collective profits. This cooperative decision-making has been demonstrated using entangled photons^31–33^, instead of single photons. In this implementation, the function of photodetectors, where photons are observed, corresponds to slot machine selection. However, making decisions with constant sampling is challenging due to the randomness of photon emission, and executing these decisions experimentally requires a stable environment. Hence, there is a growing need for a photonic accelerator that offers more flexible scalability and better experimental feasibility for cooperative decision-making.

Given the need for experimental feasibility, decision-making strategies utilizing chaotic lasers have been implemented using learning thresholds and temporal waveforms produced by semiconductor lasers with feedback^18^. Chaotic laser systems, compared to single-photon systems, are more conducive to large-scale experiments with fiber optics, such as physical random number generators^34,35^. However, decision-making using chaotic waveforms has been limited to the MAB problem and has not been explored for the CMAB problem. This approach does not exploit the inherent properties of the physical system during exploration. This is because the exploration is carried out by simply adjusting a preset threshold, with no alterations occurring in the optical system throughout this process. Therefore, we shifted our focus to the synchronization phenomena of chaotic lasers. Chaotic lasers not only generate rapid and complex temporal waveforms but also exhibit synchronization phenomena. These synchronized behaviors have characteristics that are highly beneficial for decision-making. Various applications leveraging this synchronization have been reported, including secure communication^36^, secure key distribution^37^, and reservoir computing^7^. We have numerically and experimentally implemented a decision-making system using lag synchronization of chaos^20,38,39^. This approach expands the possibilities for practical applications of chaotic lasers in the realm of decision-making.

The key to this decision-making system is low-frequency fluctuation (LFF) dynamics^40^. The temporal waveform in LFF dynamics is characterized by rapid chaotic fluctuations on the order of GHz and periodic fluctuation frequency on the order of MHz, which consists of a sudden dropout and a gradual recovery phase^41^. Thus, LFF embodies dynamics that possess both chaotic and periodic properties. The lag synchronization of chaos refers to the phenomenon wherein one laser synchronizes with another laser, with a time delay equivalent to the propagation delay time, denoted by $\tau$. In this lag synchronization, a laser with an advanced oscillation is called the “leader”, while a laser that synchronizes with another laser is called the “laggard”. Interestingly, this leader–laggard relationship in LFF dynamics spontaneously switches with a delay time $\tau$^39^. In the decision-making system based on LFF^20^, each slot was assigned to a specific laser, and the player selected the slot corresponding to the leader laser. By switching the leader–laggard relationship and adjusting the leader probability by using coupling strengths, effective exploration was achieved, and the winning machine was correctly identified. This decision-making principle is scalable to scenarios with a multitude of slot machine options^42^. However, it is important to note that this system does not realize conflict-free joint decisions.

To equip this system with the capability of conflict-free joint decision-making, we shift our focus on another synchronous phenomenon known as zero-lag synchronization. This phenomenon occurs when the temporal waveforms of semiconductor lasers in a laser network synchronize without any time delay^42–45^. For example, in a network with three mutually-coupled lasers arranged linearly, the two outer lasers synchronize with zero lag. Meanwhile, an outer laser and an inner laser synchronize with a delay equal to the time required for light to propagate between the coupled lasers^46^. This phenomenon enables lasers that are physically distant from each other to synchronize without delay. We expect to achieve a decision-making system that is capable of making conflict-free joint decisions using zero-lag synchronization.

In this study, we propose a cooperative decision-making scheme to address the CMAB problem by utilizing a laser network that exhibits both lag synchronization and zero-lag synchronization of LFF dynamics. Similar to previous work^20,42^, decisions of each player in our system are made by leveraging the leader–laggard relationships in lasers. Initially, we propose a network for cooperative decision-making and validate its effectiveness through numerical simulations. Subsequently, we implement this network experimentally and demonstrate cooperative decision-making in action. In these sections, we investigate a basic scenario involving two players and two slot machines (2-player, 2-slot situation), and then we describe the expansion of the system and examine the numerical performance of the expanded system. The innovative aspect of this study is realizing the avoidance of selection collision among players by exploiting zero-lag synchronization. No physical conflict avoidance principle has ever been demonstrated to be so flexible and scalable, and it greatly expands the possibilities for decision-making with photonics.

## Results

**Figure 1:**
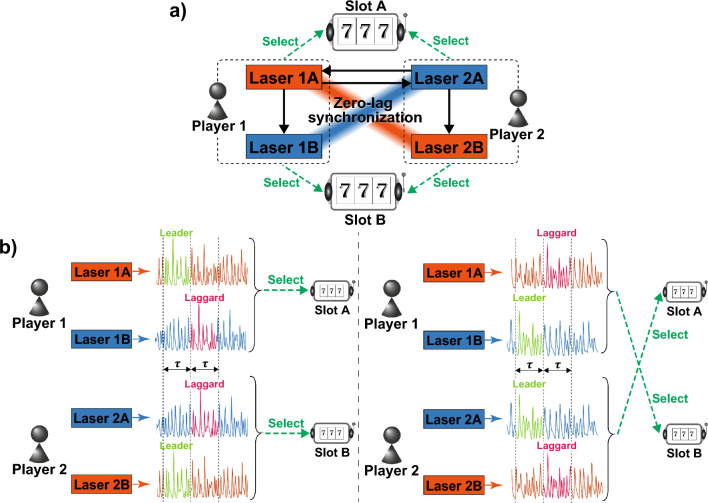
System architecture for addressing the competitive multi-armed (CMAB) problem in a 2-player, 2-slot context. (**a**) Schematic diagram of decision-making using zero-lag synchronization in a laser network. (**b**) Players’ decision on slot machine selection facilitated by lag synchronization. The zero-lag synchronization enables conflict-free joint decision-making between Players 1 and 2.

### Decision-making method and network configuration

In this research, we focus on the CMAB problem, which involves multiple players selecting from multiple slots with unknown hit probabilities in order to maximize their profit. Each slot is assumed to pay a constant reward of 1 according to static probability distributions. However, if multiple players choose the same slot machine, the rewards are divided among them. Consequently, players must solve the CMAB problem using a certain scheme as the MAB problem while avoiding selection conflicts. First, we consider the decision-making process for two players choosing between two slot machines. By examining this fundamental situation, we aim to demonstrate a new cooperative decision-making principle. We believe that this novel principle can be applied to a variety of problems.

Figure 1a illustrates the principle of decision-making for a 2-player, 2-slot situation using a laser network. In this system, Lasers 1A and 1B are assigned to Player 1, while Lasers 2A and 2B are assigned to Player 2. Players make decisions by utilizing the lag synchronization of chaos in two lasers assigned to them. At the same time, Lasers 1A and 2A are assigned to Slot A, and Lasers 1B and 2B are assigned to Slot B. Based on the leader–laggard relationship in lag synchronization of chaos, players select a slot corresponding to the leader laser among the lasers assigned to each of them. For instance, if Laser 1A becomes the leader between Lasers 1A and 1B, Player 1 selects Slot A. Conversely, if Laser 2B becomes the leader between Lasers 2A and 2B, Player 2 selects Slot B.

To achieve cooperative decision-making, zero-lag synchronization in the laser network helps avoid selection collisions among players. The laser network in Fig. 1a comprises one set of two mutually-coupled lasers (Lasers 1A and 2A) and two lasers injected from the mutually-coupled lasers (Lasers 1B and 2B). Lasers 1B and 2B are expected to synchronize with Lasers 2A and 1A, respectively, because Lasers 1B and 2B are injected from Lasers 1A and 2A, respectively, similar to Lasers 2A and 1A. Theoretically, we can find clusters of lasers in the laser network that synchronize with zero-lag using an adjacency matrix^42,43^. In general, each element in an adjacency matrix raised to the *n*-th power provides how many paths of length *n* from the row to the column are there. In addition, by applying the sign function to the power of the adjacency matrix of the laser network, each column in this matrix reveals light injection from itself or other lasers. The adjacency matrix of laser network (*G*) in Fig. 1a is as follows:

1 $$\begin{aligned} G=\begin{bmatrix} 0&{}1&{}1&{}0\\ 0&{}0&{}0&{}0\\ 1&{}0&{}0&{}1\\ 0&{}0&{}0&{}0\\ \end{bmatrix}. \end{aligned}$$

Each row and column of *G* represents Laser 1A, 1B, 2A, and 2B in order. In this case, there are only two types of adjacency matrix *G* to any *n*-th power with the sign function applied as follows $(k\in {\mathbb {N}})$:

2 $$\begin{aligned} {\textrm{sign}}(G^n)= {\left\{ \begin{array}{ll} \begin{bmatrix} 0&{}1&{}1&{}0\\ 0&{}0&{}0&{}0\\ 1&{}0&{}0&{}1\\ 0&{}0&{}0&{}0\\ \end{bmatrix} &{} (n=2k-1),\\ \begin{bmatrix} 1&{}0&{}0&{}1\\ 0&{}0&{}0&{}0\\ 0&{}1&{}1&{}0\\ 0&{}0&{}0&{}0\\ \end{bmatrix}&(n=2k). \end{array}\right. } \end{aligned}$$

From this result, Lasers 1A and 2B can synchronize with zero-lag because the elements of the first and fourth columns are the same at any time. At the same time, Lasers 1B and 2A can synchronize with zero-lag because the elements of the second and third columns are the same. Therefore, when Player 1 determines that Laser 1A is the leader, Player 2 is expected to determine that Laser 2B is the leader. Likewise, when Player 1 determines that Laser 1B is the leader, Player 2 is expected to determine that Laser 2A is the leader. These clusters of zero-lag synchronization help to avoid selection conflicts, as shown in Fig. 1b.

### Numerical simulations

We numerically simulated the performance of this network and the cooperative decision-making. We modeled the behavior of coupled semiconductor lasers using the Lang–Kobayashi equation as follows^47^ $(j=\textrm{1A, 1B, 2A, 2B})$:

3 $$\begin{aligned} \frac{dE_{j}(t)}{dt}&=\frac{1+i\alpha }{2}\left[ \frac{G_N[N_{j}(t)-N_0]}{1+\epsilon |E_{j}(t)|^2}-\frac{1}{\tau _p}\right] E_{j}(t)+\kappa E_{g(j) }(t-\tau )\exp [i\theta _{j}(t)], \end{aligned}$$

4 $$\begin{aligned} \frac{dN_j(t)}{dt}&=J-\frac{N_j(t)}{\tau _s}-\frac{G_N[N_j(t)-N_0]}{1+\epsilon |E_j(t)|^2}|E_j(t)|^2, \end{aligned}$$

5 $$\begin{aligned} \theta _j(t)&=(\omega _{g(j)}-\omega _j)t-\omega _{g(j)}\tau , \end{aligned}$$

where $E_j(t)$, $N_j(t)$, and $\theta _j(t)$ are the complex electric-field amplitude, the carrier density, and the optical phase difference between injected light and simplex oscillation light of Laser *j*, at time *t*, respectively. The second part of Eq. (3) is about the effect of injection light. Laser *g*(*j*) injects light into Laser *j*. In this network, *g*(*j*) is configured as follows:

6 $$\begin{aligned} g(j)= {\left\{ \begin{array}{ll} \textrm{2A} &{} (j=\textrm{1A}), \\ \textrm{1A} &{} (j=\textrm{1B}), \\ \textrm{1A}&{}(j=\textrm{2A}), \\ \textrm{2A}&{}(j=\textrm{2B}). \end{array}\right. } \end{aligned}$$

$\kappa$ represents the coupling strength between lasers, and $\tau$ denotes the propagation delay times of an injection path. In this simulation, $\kappa$ is set to 30 ns^-1^, and $\tau$ is set to 5 ns. These factors are critical in understanding synchronization delay and the period of leader–laggard switching, which are important quantities characterizing the system’s behavior. In this simulation, all detuning at the initial optical frequency is set to 0 Hz to demonstrate the principle of conflict-free operation using the laser network. Other parameter values used are summarized in Table 1 in the Methods section.

**Figure 2:**
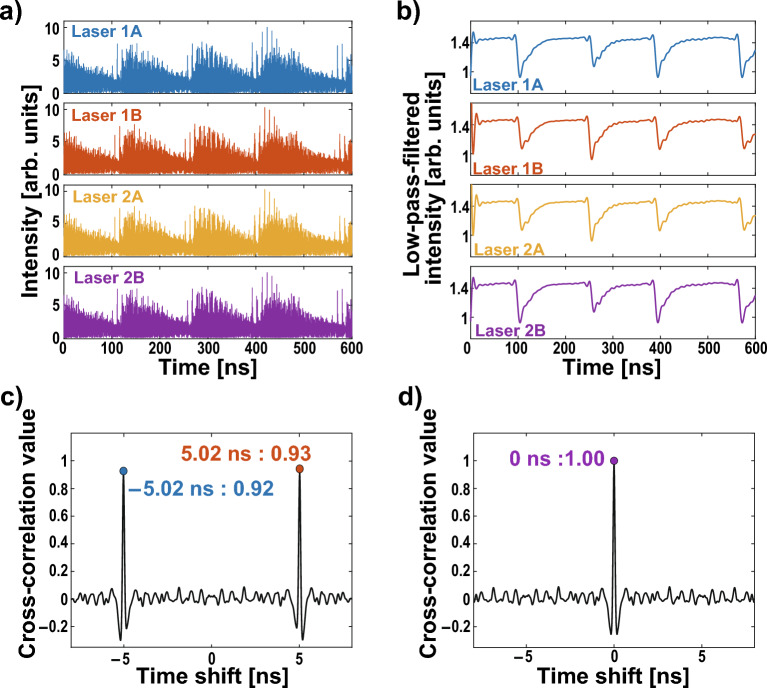
Numerical simulation results: about temporal waveforms and cross-correlation values. (**a**) Temporal waveforms of laser intensities determined by the Lang–Kobayashi equations. (**b**) Low-pass filtered temporal waveforms of laser intensity. (**c**) Cross-correlation value between Lasers 1A and Laser 1B. (**d**) Cross-correlation value between Lasers 1A and Laser 2B.

The temporal waveform of laser intensity $I_j(t) = |E_j(t)|^2$ was calculated using the Lang–Kobayashi equation. Figure 2 displays the laser intensity $I_j$, low-pass-filtered intensity, and cross-correlation value between Laser 1A–2B and Laser 1A–1B. In Fig. 2a, all lasers exhibit chaotic oscillations, but the envelope components present periodic temporal waveforms. We then examine the low-pass-filtered intensity, as shown in Fig. 2b. The low-pass filter is applied to extract periodic fluctuation on the order of MHz excluding rapid chaotic fluctuation on the order of GHz. The cutoff frequency is set at 60  MHz. Filtered intensities for all lasers demonstrate dropout and gradual recovery, indicating that the lasers oscillate due to LFFs. Furthermore, their temporal waveforms are strikingly similar.

The cross-correlation value is defined as follows (with $i,j\in \{$1A, 1B, 2A, 2B$\}$):

7 $$\begin{aligned} {\hat{C}}_{i,j}(\tau )=\frac{\langle [I_{i}(t-\tau )-\bar{I}_{i}][I_{j}(t)-\bar{I}_{j}]\rangle _T}{\sigma _{i}\sigma _{j}}, \end{aligned}$$

where $I_i$ represents the laser intensity of Laser *i*. Additionally, $\langle \cdot \rangle _T$ denotes the average over the period $T=$ 1000 ns. ${\bar{I}}_j$ and $\sigma _j$ are the averaged $I_j$ and the standard deviation of $I_j$ over the period *T*, respectively. In Fig. 2c, the cross-correlation value ${\hat{C}}_{1A,1B}$ between Lasers 1A and 1B exhibits peaks at − 5.02 ns and 5.02 ns, corresponding to the lag synchronization. Conversely, the cross-correlation value ${\hat{C}}_{1A,2B}$ between Lasers 1A and 2B has the peak at 0 s, as shown in Fig. 2d, meaning that Lasers 1A and 2B are zero-lag synchronized. From these results, it is numerically shown that zero-lag synchronization occurs as intended. We also observe that Lasers 2A and 2B exhibit lag synchronization of chaos, while Lasers 1B and 2A are in the zero-lag synchronization. Therefore, this laser network allows for the coexistence of lag synchronization chaos and zero-lag synchronization.

We focus on the leader–laggard relationship in the lag synchronization of chaos. The leader–laggard relationship is quantified using the short-term cross-correlation (STCC) value as follows:

8 $$\begin{aligned} C_{\textrm{1A}}(t)&=\frac{\langle [I_{\textrm{1B}}(t-\tau )-\bar{I}_{\textrm{1B}}][I_{\textrm{1A}}(t)-\bar{I}_{\textrm{1A}}]\rangle _\tau }{\sigma _{\textrm{1A}}\sigma _{\textrm{1B}}}, \end{aligned}$$

9 $$\begin{aligned} C_{\textrm{1B}}(t)&=\frac{\langle [I_{\textrm{1A}}(t-\tau )-\bar{I}_{\textrm{1A}}][I_{\textrm{1B}}(t)-\bar{I}_{\textrm{1B}}]\rangle _\tau }{\sigma _{\textrm{1A}}\sigma _{\textrm{1B}}}, \end{aligned}$$

10 $$\begin{aligned} C_{\textrm{2A}}(t)&=\frac{\langle [I_{\textrm{2B}}(t-\tau )-\bar{I}_{\textrm{2B}}][I_{\textrm{2A}}(t)-\bar{I}_{\textrm{2A}}]\rangle _\tau }{\sigma _{\textrm{2A}}\sigma _{\textrm{2B}}}, \end{aligned}$$

11 $$\begin{aligned} C_{\textrm{2B}}(t)&=\frac{\langle [I_{\textrm{2A}}(t-\tau )-\bar{I}_{\textrm{2A}}][I_{\textrm{2B}}(t)-\bar{I}_{\textrm{2B}}]\rangle _\tau }{\sigma _{\textrm{2A}}\sigma _{\textrm{2B}}}, \end{aligned}$$

**Figure 3:**
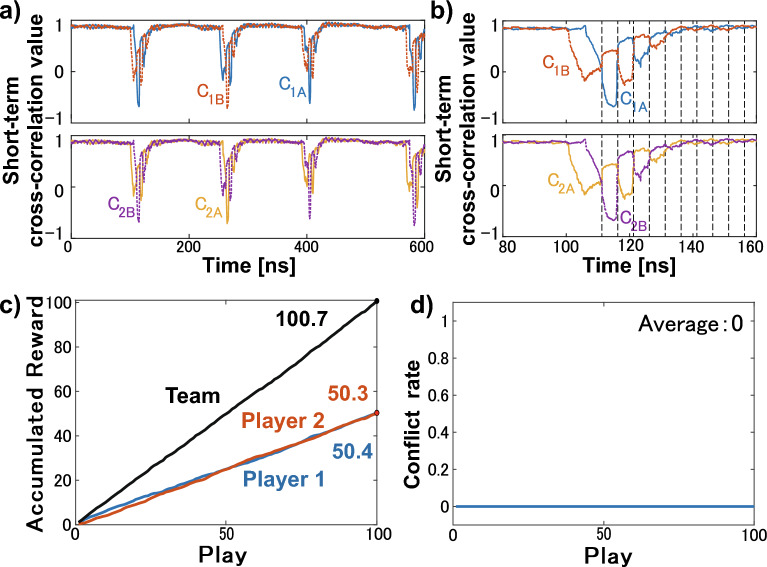
Numerical simulation results: short-term cross-correlation values and decision-making. (**a**) Short-term cross-correlation values $C_{1A}$ (blue curve), $C_{1B}$ (orange curve), $C_{2A}$ (yellow curve), and $C_{2B}$ (purple curve) calculated from Fig. 2a. (**b**) Enlarged view of short-term cross-correlation values. (**c**) Accumulated rewards for Player 1 (blue curve), Player 2 (orange curve), and the team (black curve) in the decision-making. (**d**) Conflict rate between Players 1 and 2 in decision-making.

where $\langle \cdot \rangle _\tau$ denotes the short-term average over the period $\tau$. ${\bar{I}}_j$ and $\sigma _j$ represent the averaged $I_j$ and the standard deviation of $I_j$ over the period $\tau$, respectively. Note that the short-term cross-correlation value has time *t* as a parameter, rather than the delay time $\tau$, which is used in the cross-correlation value. By employing STCC values, we can observe the changes in cross-correlation values between lasers assigned to each player as a function of time. $C_{\textrm{1A}}(t)$ represents the cross-correlation value when Laser 1A is considered as the laggard over the short-term period $\tau$, while $C_{\textrm{1B}}(t)$ considers Laser 1B as the laggard. Consequently, at time *t*, if $C_{\textrm{1A}}$ is smaller than $C_{\textrm{1B}}$, Laser 1A is the leader and Laser 1B is the laggard; the reverse is also true. Similarly, $C_{2A}$ and $C_{2B}$ reveal the relationships between Lasers 2A and 2B.

Using this temporal waveform in Fig. 2a, we calculate the STCC values and perform decision-making numerically. Figure 3 displays the STCC values and decision-making results. Comparing Figs. 2b and 3a, it can be observed that the LPF and STCC correspond closely, particularly during the dropout and recovery processes. Figure 3b presents an enlarged view of Fig. 3a. Despite the one-way coupling between Lasers 1A and 1B, STCC values $C_{1A}$ and $C_{1B}$ spontaneously switch every 5 ns, corresponding to the coupling delay time, as seen in lag synchronization of chaos. Moreover, STCC values $C_{1A}$ and $C_{1B}$ are the same as $C_{2B}$ and $C_{2A}$, respectively, since each laser is synchronized with zero-lag (Laser 1A–2B and Laser 1B–2A). Decision-making is performed using the switching of these correlation values. The entire STCC values obtained from the waveforms were used in chronological order. The sampling interval for players’ selecting slot machines is set to 1 ns, equivalent to 1 play. We expect that the selection of each player will switch without selection conflict every 5 plays because the coupling delay time is set to 5 ns. The hit probabilities of Slots A and B are set to 0.8 and 0.2, respectively. Since exploration is out of scope in this study, these hit probabilities are not very meaningful in terms of exploration. However, we set the probabilities to such biased values to ensure that the selection switch occurs ideally and players select slots equally. We test the cooperative decision-making for 100 plays. We define 100 plays as one cycle and evaluate the average accumulated reward for 10 cycles. Figure 3c displays the averaged accumulated reward, while Fig. 3d displays the averaged decision conflict rate over 10 cycles. In this problem, the team’s average theoretical maximum reward of 100 can be achieved if no selection conflict occurs. In Fig. 3c, the accumulated rewards of Players 1 and 2 are 50.4 and 50.3, and that of the team is 100.7. Also, no conflict occurs in 10 cycles, as shown in Fig. 3c. Thus, using this network, cooperative decision-making is successfully achieved, and the reward for individuals and the team reaches the theoretical maximum reward. In addition, players are rewarded equally, even though the hit probabilities of slots are biased. This equality is attained because the selections of players are constantly switched, providing equal opportunities for players to choose the best slot. The accumulated rewards are an average of 10 cycles, ensuring consistency in the number of cycles between the numerical simulation and experiment. so they are slightly different from the expected values, but by increasing the number of cycles, the individual rewards are expected to converge to 50 and the team rewards to 100.

**Figure 4:**
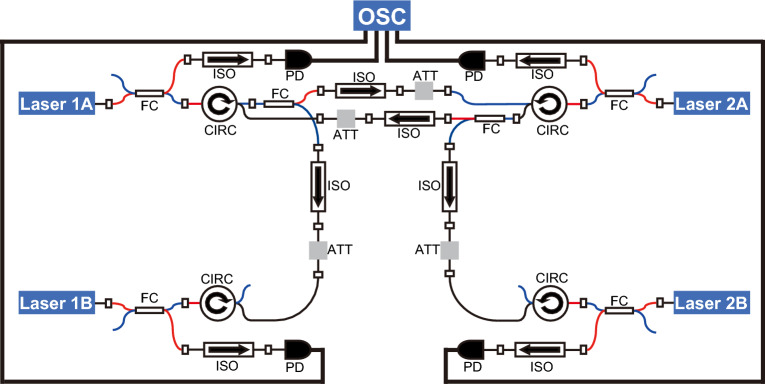
Experimental setup of the proposed decision-making system. ATT: voltage-controlled variable attenuator, CIRC: optical circulator, ISO: optical isolator, FC: fiber coupler, OSC: oscilloscope, PD: photodetector.

### Experimental results

#### Conflict-free joint decision by two players

We implement the experimental setup for the 2-player, 2-slot situation as shown in Fig. 4 to confirm the experimental possibility of our system for this fundamental situation. We use four distributed-feedback semiconductor lasers, referred to as Laser 1A, Laser 1B, Laser 2A, and Laser 2B (threshold injection current values are 11.0 mA, 11.7 mA, 11.8 mA, 11.8 mA, respectively). The light from each laser is split into a network part and a detection part by a fiber coupler. In the detection part, the light is transferred to an electrical signal by a photoreceiver and sent to the oscilloscope. In the network part, the four lasers are connected via unidirectional injection paths with optical isolators, fiber couplers, and optical circulators to configure the network in Fig. 4. Each path is adjusted to have the same delay time of 75.37 ns. Circulators on the Laser 1B and 2B sides are inserted only to make all optical injection paths the same length. In addition, a voltage-controlled variable attenuator is inserted to adjust the coupling strength $\kappa$. $\kappa _{i,j}$ is the coupling strength of the injection path from Laser *i* to Laser *j*. In order to achieve lag synchronization of chaos in the LFF dynamics, the injection current of each laser is set to about 1.1 times the threshold injection current, and the temperature is set so that the peak wavelength is 1547.100 nm. In this network, mutually coupled Lasers 1A and 2A cause LFF dynamics. Lasers 1A and 2A are also transmitted to Lasers 1B and 2B, respectively, with the delay, Lasers 1A and 2B (1B and 2A) synchronize without delay. Therefore, the coupling strengths between Lasers 1A and 2A are set to $\kappa _{1A,2A}=0.67$ and $\kappa _{2A,1A}=0.73$ to oscillate in LFF dynamics. On the other hand, the coupling strengths between Lasers 1B and 2B are set to $\kappa _{1A,1B}$ and $\kappa _{2A,2B}=1.0$ to achieve the lag synchronization.

**Figure 5:**
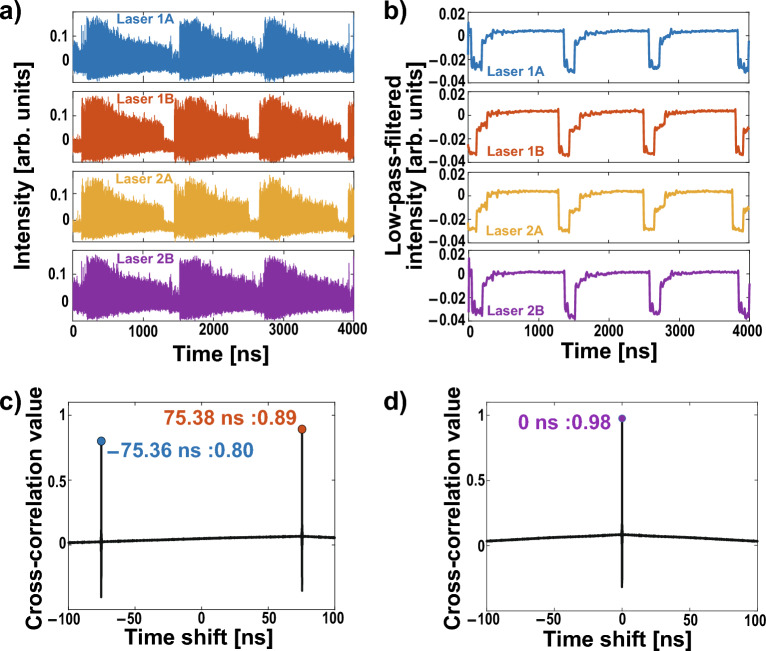
Experimental results: temporal waveforms and cross-correlation values. (**a**) Temporal waveforms of laser intensities. (**b**) Low-pass filtered temporal waveforms of laser intensities. (**c**) Cross-correlation value between Lasers 1A and Laser 1B (${{\hat{C}}}_{1A,1B}(0)$). (**d**) Cross-correlation value between Lasers 1A and Laser 2B (${{\hat{C}}}_{1A,2B}(0)$).

**Figure 6:**
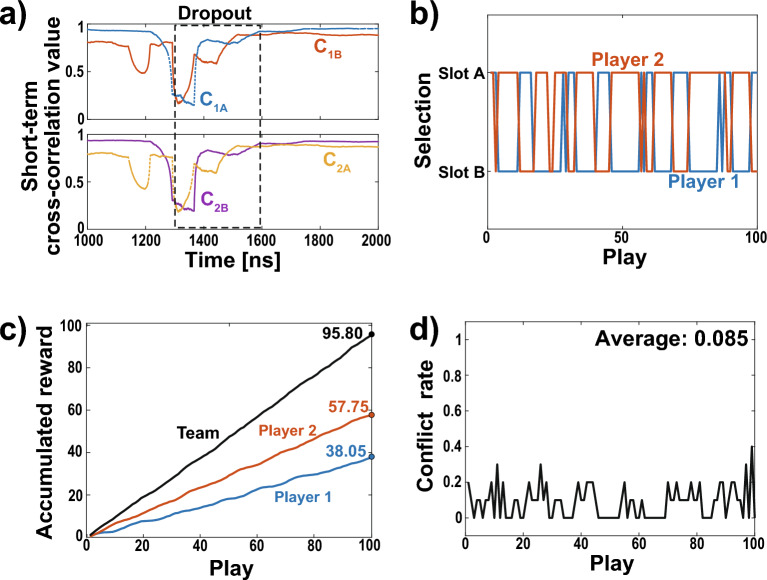
Experimental results: short-term cross-correlation values and decision-making. (**a**) Enlarged view of short-term cross-correlation values $C_{1A}$ (blue curve), $C_{1B}$ (orange curve), $C_{2A}$ (yellow curve), and $C_{2B}$ (purple curve) calculated from Fig. 5a. (**b**) Selection of Player 1 (blue curve), Player 2 (orange curve), and the team (black curve) in the decision-making. (**c)** Averaged accumulated reward of Player 1 (blue curve), Player 2 (orange curve), and team (black curve) in the decision-making. (**d**) Averaged conflict rate between Player 1 and 2 in the decision-making.

Figure 5 displays the waveforms and cross-correlation (CC) values for the laser intensities in the experiment. Figure 5a shows the temporal waveforms, showing that the lasers oscillate chaotically with the long periods. In this figure, Laser 1A (1B) is similar to Laser 2B (2A) in the range of small oscillation amplitudes. Figure 5b shows the low-pass-filtered temporal waveforms. The cutoff frequency of the low-pass filter is 60  MHz. Comparing this with Fig. 5a, Laser 1A (1B) clearly synchronizes with Laser 2B (2A). Then, we check the cross-correlation values of Lasers 1A–1B (Fig. 5c) and Lasers 1A–2B (Fig. 5d). In Fig. 5c, there are two peaks near 75.37 ns, corresponding to the propagation delay time of the experimental setup. In fact, there are differences in the actual lengths of the injection paths, and the difference of the propagation delay time is within approximately 1 ns. To compensate for these differences, the time positions of waveforms are adjusted using the oscilloscope’s deskew feature. Details of how the deskew is adjusted are described in previous research^42^. Although the positions of peaks are adjusted near 75.37 ns, there are still a few differences between delays because of the resolution of the oscilloscope. In Fig. 5d, the CC value reaches 0.98 in the case of no delay. Then, Lasers 1A and 2B are regarded as achieving zero-lag synchronization.

Using this temporal waveform in Fig. 5a, we calculate the STCC values and perform a decision-making experiment. Figure 6a shows the STCC values. Unlike the case of numerical calculation, the switching between the STCC values occurs in the dropout part. However, leader–laggard switching is crucial to achieve equal rewards among players and effective exploration. Thus, in the experimental system, we focus on regions near the dropout where switching took place. As shown in Fig. 6a, dropout is determined based on STCC values. Decision-making begins when all of the STCC values $C_{1A}$, $C_{1B}$, $C_{2A}$, and $C_{2B}$ drop below 0.4 and stops when any one of the STCC values rises above 0.94. The decision-making is made every 15.06 ns, 10 cycles each consisting of 100 plays are performed, similar to the numerical simulation. Since the propagation delay time is about five times the decision-making period, player selections are expected to switch every 5 plays. For this decision-making experiment, a waveform of approximately 15 μs is needed. Figure 6b displays the selection of each player in one cycle, while Fig. 6c presents the averaged accumulated rewards of each player and the team. Figure 6d demonstrates the averaged conflict rate between players in 10 cycles. In Fig. 6b, players mostly select different slot machines. Then, the accumulated rewards of Player 1, Player 2, and the team are 38.05, 57.75, and 95.8, respectively, and the average conflict rate is 0.085. We can see that the experimental results support the results of numerical simulations; however, there is a selection conflict, and the reward is reduced accordingly. This is because the experiments include noise, and zero-lag synchronization is not perfectly achieved. Based on these results, the cooperative decision-making system can successfully reduce the conflict rate and increase rewards for the players and the team numerically and experimentally.

**Figure 7:**
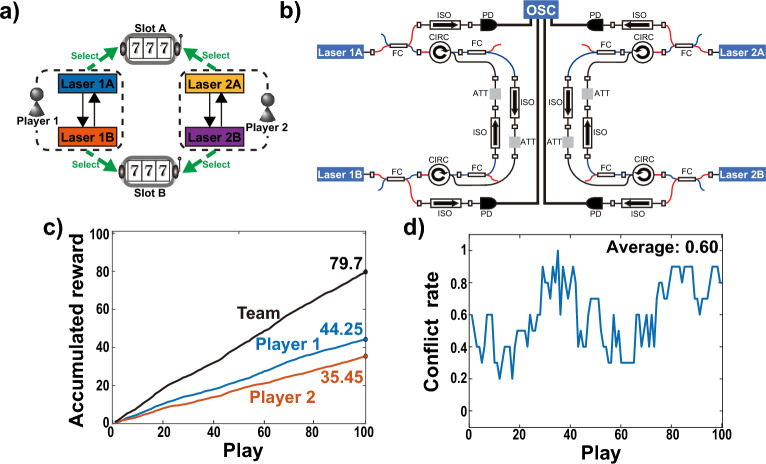
Comparative experiment description and result. (**a**) Schematic diagram illustrating decision-making using the two independent pairs of mutually coupled lasers. (**b**) Experimental setup of the comparative experiment. (**c**) Averaged accumulated rewards of Player 1 (blue curve), Player 2 (orange curve), and team (black curve) during the decision-making process. (**d**) Averaged conflict rate in the decision-making.

#### Decision-making by independent two-players

As a comparative experiment, the decision-making system is tested using two pairs of independently mutually coupled lasers, as shown in Fig. 7a,b. Laser 1A–1B and Laser 2A–2B are mutually coupled independently through unidirectional injection paths. Player 1 is assigned Lasers 1A and 1B, while Player 2 is assigned Lasers 2A and 2B. Lasers 1A and 2A are assigned to Slot A, and Lasers 1B and 2B are assigned to Slot B, similar to the system in Fig. 1a. This setup represents a situation where players make decisions independently. As shown in Fig. 7b, each injection path is composed of circulators, a fiber coupler, an attenuator, and an isolator. The fiber couplers are inserted to match the injection path lengths to the experimental setup in Fig. 4. The coupling strengths for the mutually coupled lasers, $\kappa _{1A,1B}$, $\kappa _{1B,1A}$, $\kappa _{2A,2B}$, and $\kappa _{2B,2A}$ are set to 0.339, 0.97, 0.96, 0.67, respectively.

In the comparative experiment with independent decision-making, the coupling strengths are set to generate LFF dynamics, and the biased pairs of coupling strengths compensate for the differences in the characteristics of semiconductor lasers to achieve balanced leader probability for each laser. In this scenario, the temporal waveforms in LFFs of the two lasers assigned to each player are synchronized with a delay equal to the propagation delay time, but there is no observed correlation between the lasers assigned to Player 1 and those assigned to Player 2. Using the lag synchronization of chaos, the decision-making experiment is performed similarly to the previous cooperative decision-making experiment. Figure 7c displays averaged accumulated rewards for each player and the team, and Fig. 7d presents the averaged conflict rate between players across 10 cycles. The averaged accumulated rewards of Player 1, Player 2, and the team are 35.45, 44.25, and 79.7, respectively. The averaged conflict rate is 0.60. Comparing the results of the independent decision-making system with those of the cooperative decision-making system, it becomes evident that the cooperative approach outperforms the independent one. In particular, zero-lag synchronization functions as a conflict-free joint decision in the decision-making system. The cooperative decision-making system achieves a higher team reward and a significantly lower conflict rate, highlighting its effectiveness in improving decision-making processes and outcomes.

## Discussion

### Performance and stability

In these results, numerical and experimental results are similar and are superior to the independent system. However, there is still a gap between the numerical and the experimental results of our decision-making system. First, perfect conflict-free decision-making is not achieved in the experimental setup. Although the achieved conflict rate was 0 in the numerical simulation, the averaged conflict rate in the experimental setup is about 8.5%. Second, perfect equality is not achieved in the experimental setup. It is difficult in the experiment to observe the ideal switching of the leader–laggard relationship seen in a numerical simulation. In the experimental setup, the switching occurs only around dropout, and the leader probability is biased. Therefore, using the whole waveform similar to the numerical simulation makes the reward of players biased. Even when we use near the dropout to try to achieve uniform selection switching, the averaged accumulated rewards of Player 1 and Player 2 are 38.0 and 57.7, respectively, making it difficult to guarantee adequate equality.

These deviations between the numerical and experimental results can be attributed to small differences in parameters. In the numerical simulation, the internal parameters of lasers, the propagation delay time $\tau$ of all injection paths and many other parameters comprising the network are precisely the same. This matching of parameters makes the synchronous state more stable and the system performance more ideal. Allowing small differences in these parameters, perfect conflict avoidance cannot be achieved, and a few conflicts may occur. The deterioration of the synchronization status can also make the lasers’ dynamics unstable. Some degree of misalignment in the inner parameters of lasers can be compensated by adjusting the injection current and temperature of the lasers or the coupling strength of injection paths. In this study, we adjusted the coupling strength, injection current, and initial optical frequencies of the lasers to improve synchronization and achieve an equal leader probability. However, the parameter deviations were not completely filled. Especially, we suspect that the difference in $\tau$ may be particularly significant. In other words, misalignment of $\tau$ among injection paths presumably works critically on the performance and stability of the system because the misalignment in $\tau$ causes a misalignment of arriving light to the lasers that should be synchronized. Then, using elaborate delay lines in the adjustment of $\tau$, although this experiment is conducted with deskew, can improve performance and stability.

Additionally, other networks can achieve the same synchronous state as our network. For example, a four-laser network with connections between any adjacent pairs of lasers^45^ can theoretically and numerically perform just as well as our network. However, it becomes increasingly challenging in the experiment to match the $\tau$ of all injection couplings as the number of coupling increases. Thus the number of injection couplings should be minimized. Our network has the minimum number of injection couplings. It can be expanded to situations with more players or slots while maintaining the minimum number of injection couplings.

### Scalability

Our network can be expanded to accommodate more players and more slots. An adjacency matrix corresponding to the situation that has the same number of players and slots (*n* players and *n* slots) is the $n^2\times n^2$ matrix as shown below:

12 $$\begin{aligned} G=\begin{bmatrix} A&{}O&{}O&{}&{}O&{}B\\ B&{}A&{}O&{}\dots &{}O&{}O\\ O&{}B&{}A&{}&{}O&{}O\\ &{}\vdots &{}&{}\ddots &{}\vdots \\ O&{}O&{}O&{}\dots &{}A&{}O\\ O&{}O&{}O&{}\dots &{}B&{}A\\ \end{bmatrix} \end{aligned}$$

where the matrixes *A* and *B* are the $n\times n$ matrixes as shown below:

13 $$\begin{aligned} A=\begin{bmatrix} 0&{}1&{}0&{}\dots &{}0\\ 0&{}0&{}1&{}&{}0\\ &{}\vdots &{}&{}\ddots &{}\vdots \\ 0&{}0&{}0&{}\dots &{}1\\ 0&{}0&{}0&{}&{}0\\ \end{bmatrix}; B=\begin{bmatrix} 1&{}0&{}0&{}\dots &{}0\\ 0&{}0&{}0&{}&{}0\\ &{}\vdots &{}&{}\ddots &{}\vdots \\ 0&{}0&{}0&{}\dots &{}0\\ 0&{}0&{}0&{}&{}0\\ \end{bmatrix}. \end{aligned}$$

Similarly, the network can be expanded for different numbers of players and slots.

**Figure 8:**
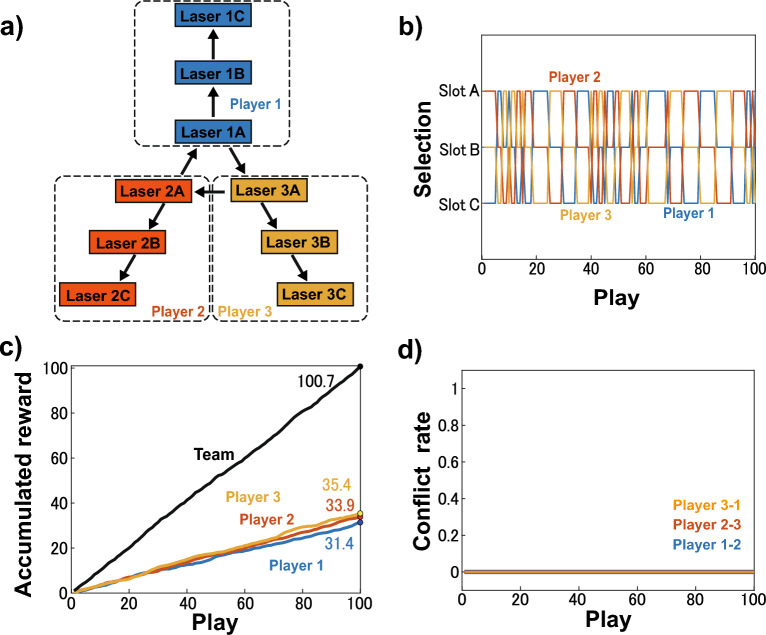
Scalability investigation. (**a**) Network configuration of the decision-making for three-player, three-slot situation. (**b**) Selections of Player 1 (blue curve), Player 2 (red curve), and Player 3 (orange curve) in the decision-making. (**c**) Averaged accumulated rewards of Player 1 (blue curve), Player 2 (red curve), Player 3 (orange curve), and team (black curve) in the decision-making. (**d**) Averaged conflict rate between Players 1 and 2 (blue curve), Players 2 and 3 (red curve), and Players 3 and 1 (orange curve) in the decision-making.

As an example of expanding the situation, the network corresponding to the expanded three-player, three-slot situation is shown in Fig. 8a. We simulate the decision-making for this expanded situation numerically. In this network, Laser *j*A, Laser *j*B, and Laser *j*C are assigned to Player *j* ($j=1, 2, 3$), and Laser 1*k*, Laser 2*k*, and Laser3*k* are assigned to Slot *k* ($k=$A, B, C). In this network, the selection of each player would be decided based on leader–laggard relationships among each player’s three lasers, like a ring configuration. Decision-making by a laser network in a ring configuration has already been established^42^. Referring to this, the STCC in lasers assigned to each player is defined similarly to Eqs. (14)–(16) as follows $(j=1, 2, 3)$:

14 $$\begin{aligned} C_{j\textrm{A}}(t)&=\frac{\langle [I_{j\textrm{C}}(t-\tau )-\bar{I}_{j\textrm{C}}][I_{j\textrm{A}}(t)-\bar{I}_{j\textrm{A}}]\rangle _\tau }{\sigma _{j\textrm{C}}\sigma _{j\textrm{A}}}, \end{aligned}$$

15 $$\begin{aligned} C_{j\textrm{B}}(t)&=\frac{\langle [I_{j\textrm{A}}(t-\tau )-\bar{I}_{j\textrm{A}}][I_{j\textrm{B}}(t)-\bar{I}_{j\textrm{B}}]\rangle _\tau }{\sigma _{j\textrm{A}}\sigma _{j\textrm{B}}}, \end{aligned}$$

16 $$\begin{aligned} C_{j\textrm{C}}(t)&=\frac{\langle [I_{j\textrm{B}}(t-\tau )-\bar{I}_{j\textrm{B}}][I_{j\textrm{C}}(t)-\bar{I}_{j\textrm{C}}]\rangle _\tau }{\sigma _{j\textrm{B}}\sigma _{j\textrm{C}}}. \end{aligned}$$

$C_{jk} (k=\textrm{A}, \textrm{B}, \textrm{C})$ represents the cross-correlation value when Laser *jk* is considered as the laggard over the short-term period $\tau$, for example, $C_{\textrm{1B}}(t)$ considers Laser 1B lagging behind Laser 1A. Consequently, among lasers assigned to each player, the one having the minimum STCC value is the leader laser. For example, if $C_{\textrm{1A}}$ is smaller than $C_{\textrm{1B}}$ and $C_{\textrm{1C}}$, Laser 1A is the leader among Lasers 1A, 1B, and 1C, and other cases are also true.

Parameter values used are summarized in Table 1 in the Methods section. Players’ selections are every 1 ns, which is the same as the numerical simulation, corresponding to 5 identical selections in the duration of the switching of the leader. In each cycle, Slots A, B, and C payout rewards of 1 with probabilities of 0.6, 0.3, and 0.1, respectively. Figure 8b displays the selection made by each player in one cycle, while Fig. 8c displays the averaged accumulated reward of each player and team, and Fig. 8d displays the averaged conflict rate between players over 10 cycles. The conflict among the three players is completely avoided, and team or player rewards are maximized. By switching selections of players, each player receives rewards almost equally, although the probabilities of slots are biased. If three players make decisions independently, the expected conflict rate is about 77%, and the expected rewards of the team and each player are about 56 and 18, respectively. The expected conflict rate would grow, and the expected rewards of the team would decrease with independent selection as the number of players increases. Thus, the effect of this conflict-free principle is also more significant in expanded situations.

Thus, this system works well in extended situations. As mentioned earlier, it is possible to implement this expanded system experimentally by devising a way to align the length of each injection coupling. Of course, as *n* increases, the number of lasers and injection paths required increases to $n\times n$. To alleviate the increased physical resource requirements in implementation, using surface emitting lasers or laser arrays and taking an optical integration approach can be considered. Optical integration also reduces the length of the optical injection path, which has the advantage of shortening the switching period corresponding to the transmission delay time tau, thereby reducing the time required to perform the search^20^. In addition to the cases mentioned above, we consider this system possible to extend to situations where the number of players and slots are not the same. In such a situation, combined conflict avoidance with the effective exploration demonstrated in the previous study^20^, reward maximization would be achieved.

## Conclusion

In this research, we have proposed a cooperative decision-making system and have confirmed its applicability. In the numerical simulations, the averaged accumulated rewards of Player 1, Player 2, and the team are 50.4, 50.3, and 100.7, respectively. When the players make decisions fully cooperatively with no conflict, the expected reward for each player is 50, and for the team is 100. The numerical simulation achieved this theoretical maximum reward. When players make decisions independently, the expected reward for each player is 37.5, for the team is 75, and the expected conflict rate is 0.5. The conflict rate and reward improved significantly thanks to the proposed cooperative mechanism. This demonstrates the usefulness of the proposed system numerically. In the experimental setup, the averaged accumulated rewards of Player 1, Player 2, and the team are 38.0, 57.7, and 95.8, respectively, with an average conflict rate of 0.085. In the comparative experiment with independently coupled lasers corresponding to the non-cooperative decision-making, the averaged accumulated rewards of Player 1, Player 2, and the team are 35.4, 44.2, and 79.7, respectively, with an average conflict rate of 0.60. These results confirm the superiority of our system. Furthermore, this system can be expanded to situations with more players and slots. Network expansion is theoretically generalized. In the three-player, three-armed situation, perfect conflict-free decision-making is also achieved numerically, and the averaged accumulated rewards of Player 1, Player 2, Player 3, and the team are 31.4, 33.9, 35.4, and 100.7, respectively.

There are still issues that need to be resolved to fully realize our system’s numerical and theoretical excellent performance in the experimental setup. All propagation delay times of injection paths presumably should be matched more than the current situation for experimental performance and stability. We believe that this issue can be solved technically, and we expect that this system will be further expanded and realized in the experimental system in the future.

A series of studies on decision-making in laser networks has special significance in research on chaotic lasers. In many examples of applied research so far, the chaotic laser has played a role similar to a signal generator, such as in physical random number generation^34^ and secure key generation^37^, or a converter, such as in reservoir computing^7^. Therefore, the chaotic laser system was insensitive to the application in both system and dynamics. However, in decision-making using laser networks^20,42^ and multimode lasers^22^, the laser system can be directly changed by the result of decision-making. In other words, the laser system will be applied to a decision-making environment. On the other hand, photonic decision-making seems to be one of the applications of controlling chaos^48^ because decision-making controls laser systems. We hope these decision-making studies will reboot research related to controlling chaos, and usher in the dawn of adaptive physical systems in not only chaotic laser but also light research.

## Methods

### Details of numerical experiments

The parameters of the numerical simulation are described in Table 1. The parameters are determined based on references^20,39,42^. $\Delta f_{\textrm{ini}}$ refers to the initial optical frequency detuning of each laser from the frequency of Laser 1A, $f_{1A}=c/\lambda _{1A}$.

**Table 1:** Parameters of the numerical simulation.

Symbol	Parameter	Value
$G_N$	Gain coefficient	8.40 $\times$ 10^-13^ m^3^ s^-1^
$N_0$	Carrier density at transparency	1.40$\times$ 10^24^ m^3^
$\epsilon$	Gain saturation coefficient	2.0$\times$ 10^-23^
$\tau _p$	Photon lifetime	1.927 $\times$ 10^-12^ s
$\tau _s$	Carrier lifetime	2.04 $\times$ 10^-9^ s
$\alpha$	Linewidth enhancement factor	3.0
$\lambda _{1A}$	Optical wavelength of laser 1A	1.537 $\times$ 10^-6^ m
*c*	Speed of light	2.998 $\times$ 10^8^ m s^-1^
$\kappa$	Coupling strength	30.00 $\times$ 10^-9^ s^-1^
$j=J/J_{th}$	Normalized injection current	1.1
$\tau$	Propagation delay time of light	5.0 $\times$ 10^-9^ s
$\Delta f_{\textrm{ini}}$	Initial optical frequency detuning	0 Hz

### Details of experiments

Details of the experimental equipment are described in Table 2. In this research, semiconductor lasers have no isolator. This feature allows light to be injected into the laser from another laser. In our network, two types of fiber couplers are used, with light split ratios of 90:10 and 50:50. The coupler adjacent to each laser has a 90:10 split, and 10% of the lasers output is sent to the oscilloscope via an isolator and a photoreceiver. The couplers coordinating the injection paths have a 50:50 split. The type of semiconductor laser is a distributed-feedback semiconductor laser. The threshold injection current value and actual injection current value of each laser used in the experiment are shown in Table 3.

**Table 2:** Details of experimental equipment.

Component	Manufacturer	Model number	Details
Semiconductor laser	NTT electronics	NLK1C5GAAA	Wavelength of 1547.79 nm, no isolator
Photoreceiver	Thorlabs	RXM10AF	10 GHz bandwidth
Oscilloscope	Tektronix	MSO72304DX	23 GHz bandwidth, 50 G Sample/s
Voltage-controlled variable attenuator	Thorlabs	V1550PA	Wavelength of 1550 nm
Optical circulators	Thorlab	CIR1550PM-APC	Wavelength of 1520–1580 nm
Fiber couplers	Thorlabs	PN1550R5A2	Wavelength of 1550 ± 15 nm, 90:10 split
		PN1550R5A1	Wavelength of 1550 ± 15 nm, 50:50 split
Optical isolator	Thorlab	IOT-G-1550A	Wavelength of 1550 nm

**Table 3:** The threshold injection current of each laser.

Laser	Threshold injection current value (mA)	Injection current value (mA)
Laser 1A	11.0	12.0
Laser 1B	11.6	12.7
Laser 2A	11.7	12.9
Laser 2B	11.8	13.0

## Data Availability

The data that support the plots within this thesis are available from H.I. upon reasonable request.
